# Tobacco Imagery in Movies and Web Series Streaming in India and Their Compliance With the Recent Anti-tobacco Rules for Over-the-Top (OTT) Platforms

**DOI:** 10.7759/cureus.55730

**Published:** 2024-03-07

**Authors:** Pratap K Jena, Arpita Patel, Jugal Kishore, Amit Yadav, Nancy Satpathy, Soumini Samal, Aishwarya Garnaik, Manasmruti Sahu, Sagarika Das, Satyabhama Sahoo

**Affiliations:** 1 Health Care Management, Swiss School of Business and Management (SSBM) Geneva, Geneva, CHE; 2 School of Public Health, Kalinga Institute of Industrial Technology (Deemed to Be University), Bhubaneswar, IND; 3 Public Health, Kalinga Institute of Industrial Technology (KIIT) School of Public Health, Bhubaneswar, IND; 4 Dentistry, Hi-Tech Dental College and Hospital, Bhubaneswar, IND; 5 Community Medicine, Vardhman Mahavir Medical College and Safdarjung Hospital, New Delhi, IND; 6 Tobacco Control, Vital Strategies, New Delhi, IND; 7 Public Health, Indian Council of Medical Research (ICMR), New Delhi, IND; 8 Community Medicine, Siksha 'O' Anusandhan, Bhubaneswar, IND; 9 Nursing, Shri Jagdishprasad Jhabarmal Tibrewala (JJT) University, Jhunjhunu, IND; 10 Mental Health Nursing, Central Institute of Psychiatry, Ranchi, IND

**Keywords:** tobacco free films, tobacco promotion, c.o.t.p.a, o.t.t. rules 2023, tobacco imagery

## Abstract

Introduction: Internet access, smartphones, and televisions have significantly boosted over-the-top (OTT) movies and web series viewing in India, especially among youths. Despite restrictions, OTT platforms continue to promote tobacco products. India has recently enforced the revised OTT Rules 2023 effective September 1, 2023, to counter tobacco promotion in OTT shows. This study explores compliance with the OTT Rules 2023 in popular movies and web series on select OTT platforms in India.

Methods: About 29 movies and 31 web series from seven popular OTT platforms as of September 26, 2023, were analyzed in this study. The incidence of tobacco imagery and compliance with the OTT Rules 2023 were assessed using a standardized format with the help of seven trained coders. Descriptive statistics were used to describe instances of tobacco imagery and violations of the provisions of Indian law.

Results: The average incidence of tobacco imagery per included show was 3.95. None of the movies and web series fully complied with the provisions of health spots and audio-visual warnings. Only 35.7% of the shows (movies: 57.1%, web series: 14.3%) fully complied with the anti-tobacco static message provisions. The foreign-origin movies had zero compliance with static messages, though they had fewer tobacco images. Half of the shows for children up to 12 years old had tobacco imagery but fully complied with the static warning message provisions.

Conclusion: The portrayal of tobacco imagery in OTT shows is prevalent, and their poor compliance with the OTT Rules 2023 is a concern. Therefore, monitoring and stricter enforcement of the OTT Rules should be given priority to protect viewers from tobacco promotion in OTT shows.

## Introduction

Exposure to tobacco imagery in movies plays a vital role in the initiation and continuation of tobacco use, especially among youths [1], and product placement in movies by tobacco companies has made smoking a socially acceptable and attractive behavior [1, 2]. The WHO Framework Convention on Tobacco Control (FCTC) recommended limiting youth exposure to tobacco images in all forms of media, including the Internet, mobile, and new technologies [3]. Both direct and indirect tobacco advertising, promotion, and sponsorship (TAPS) have been banned under Section 5 of the Indian tobacco control law, Cigarettes and Other Tobacco Products Act (COTPA) [4].

The COTPA 2003 legislation introduced "Tobacco-free films and TV rules" in 2005 to ban tobacco image depiction in movies and TV shows. A revised regulation from 2012 required Central Board of Film Certification-approved substantial justification as a condition for the display of tobacco imagery [5, 6]. Additionally, the revised rule mandated a clear display of strict health warnings at the bottom of the screen during tobacco imagery incidences. Moreover, films must incorporate anti-tobacco audio-visual health spots lasting 30 seconds and include an audio-visual disclaimer of a minimum duration of 20 seconds regarding the harmful effects of tobacco use. These health spots and disclaimers should be displayed at the beginning of the film and during the intermission [7]. The OTT Rules 2023 is attached as a supplementary file in the appendices section for reference. Restrictions in movies and TV shows on tobacco promotions lead to a focus on tobacco promotion on popular online streaming over-the-top (OTT) platforms. As regulatory restrictions help reduce exposure to tobacco promotion [8, 9], regulation of OTT content for tobacco promotion is essential.

Minor-rated films continue to portray smoking despite prohibitions [1], which could promote tobacco use among teenagers [10, 11]. OTT content is heavily consumed in India due to mobile internet penetration and is popular among youths [12]. At the end of 2022, India was home to about 424 million OTT viewers and 119 million paid subscribers [13]. In recent times, movies, web series, OTT content, and social media have remained significant sources of exposure to tobacco imagery among adolescents [14, 15].

A concerning trend in the depiction of tobacco imagery on OTT platforms without warnings [11, 16-18] led India to bring an exclusive law, OTT Rules 2023, to regulate the promotion on OTT streaming platforms effective September 1, 2023 [19]. New rules require health spots, warnings, and disclaimers during shows; non-compliance results in action. In this context, the study explores the incidence of tobacco imagery in top-ranked movies and web series on select OTT platforms in India and assesses their compliance with the latest COTPA Amendment Rules, 2023.

## Materials and methods

**Figure 1:**
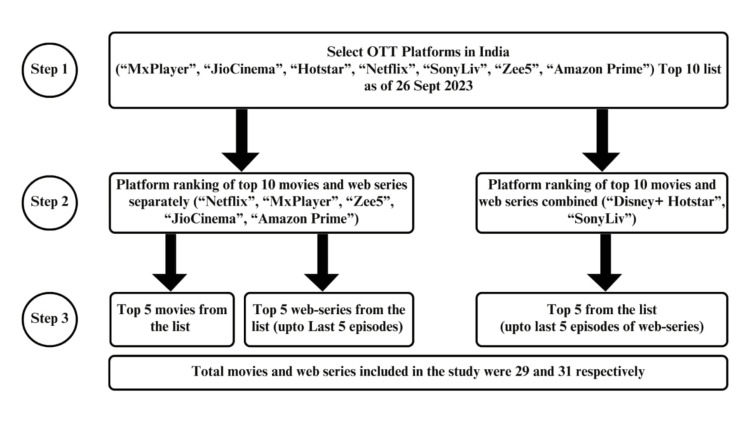
Selection Process of Movies and Web Series in the Study.

A cross-sectional research design was used to meet the study objectives. The Government of India has listed 57 OTT platforms [20]. Various agencies regularly publish a list of popular OTTs at regular intervals [21-24]. The authors narrowed down the top seven OTT platforms based on access, popularity among viewers, and consensus among authors. Each OTT platform maintains its own top 10 list; some make it for movies and series separately, and some in a single list. The selection process for movies and web series has been outlined in Figure 1. The OTT shows for this study were selected based on the platform's reported top 10 list as of September 26, 2023. The timeframe for data collection was two months (October-November, 2023). In the case of the series, only the last five episodes of the current season streaming as of September 26, 2023, were considered. A total of 29 movies and 31 web series (136 episodes) were considered for the study purpose.

The Breathe California methodology was deployed to assess tobacco imagery incidences in top movies and web series [11, 25]. A team of seven coders underwent an extensive training session covering the application of the method and a thorough understanding of tobacco-free film and TV regulations in India, in addition to the recent OTT Rules 2023. Each coder was assigned a specific OTT platform and completed individual data collection forms for every episode and movie within their allocated platforms. Each instance of tobacco imagery and violation of the OTT Rules 2023 provisions were recorded in the tool. The first and second authors verified the instances of tobacco imagery recorded by coders.

A tobacco incident was specifically outlined as the clear use of a tobacco product by an actor or actress in the visual content. Every instance where an actor or actress was shown using or handling a tobacco product was precisely counted as one tobacco incident.

To assess adherence to current tobacco-related regulations in India, the coders systematically recorded key details on the data collection forms for the selected web series and movies. The key information included was the age rating of episodes (13+, 16+, or 18+), language, country of origin, presence of anti-tobacco health spots of 30 seconds, and audio-visual disclaimer of 20 seconds duration at the beginning and middle of the program. In addition, data on the presence of a conspicuous warning message about tobacco at the bottom of the screen when displaying tobacco products were collected.

## Results

The details of OTT platforms, names of the movies and web series, country of production, category of shows, tobacco incidents, and compliance with the OTT Rules 2023 were provided in Tables 1, 2, which indicate 237 (movies: 137, series: 100) incidences of tobacco imagery in 29 movies and 31 web series. Approximately 45% of web series and 48% of movies displayed tobacco images. The summary of the tobacco incidence and violation of the OTT Rules 2023 is presented in Table 3.

**Table 1:** Tobacco incidents and compliance with OTT (over-the-top) rules, 2023, in movies in India U: Unrestricted public exhibition, suitable for all ages; UA: Unrestricted public exhibition but with caution to give parental guidance for those under 12; A: Only adults are allowed to view these programs.

OTT platform	Name of the movies	Country of production	Viewer category	Tobacco incidents	A-V disclaimer		Anti-tobacco static message		Anti-tobacco health spots	
					Shown	Language	Shown	Language	Shown	Language
MX Player	The Transporter Refueled	China, France	U/A	0	No	-	No	-	No	-
	The Expendables	US	A	0	No	-	No	-	No	-
	The November Man	UK, US	A	0	No	-	No	-	No	-
	Operation Red Sea	China	A	0	No	-	No	-	No	-
	Ekkadiki	India	U/A	0	No	-	No	-	No	-
JioCinema	Bloody Daddy	India	U/A	14	Start only	English, Hindi	Yes, always	English, Hindi	No	Not shown
	Evil Dead Rise	US	U/A	5	No	Not shown	No	Not shown	No	Not shown
	Harry Potter and Deathly Hallows Part 2	UK, US	U/A	0	No	-	No	-	No	-
	Fast X	US	U/A	0	No	-	No	-	No	-
	Vikram Vedha	India	U/A	19	No	Not shown	Yes, always	English	No	Not shown
Netflix	Jaane Jaan	India	U/A	9	Start only	English	No	Not shown	No	Not shown
	No Hard Feelings	US	A	0	No	-	No	-	No	-
	Spy Kids: Armageddon	US	U/A	0	No	-	No	-	No	-
	RDX	India	U/A	18	Start only	English	No	Not shown	No	Not shown
	Gandeevadhari Arjuna	India	U/A	1	Start only	English	No	Not shown	No	Not shown
Hotstar	Atrangi Re	India	U/A	2	Start only	Hindi	Yes, always	Hindi	YES	Hindi
	No One Will Save You	US	U/A	0	No	-	No	-	No	-
	Elemental	US	U/A	0	No	-	No	-	No	-
	Big Bull	India	U/A	0	No	-	No	-	No	-
Zee5	Tarla	India	U	0	No	-	No	-	No	-
	DD Returns	India	U/A	2	Start only	English, Tamil	Yes, always	Tamil	No	Not shown
	Hostel Hudugaru Bekagiddare	India	U/A	2	Start only	English, Kannada	No	Not shown	No	Not shown
	Haddi	India	A	9	Start only	English	No	Not shown	No	Not shown
	Dream Girl	India	U/A	0	No	-	No	-	No	-
Amazon Prime	Rocky Aur Rani Ki Prem Kahani	India	U/A	0	No	-	No	-	No	
	Jailer	India	U/A	23	Start only	English, Tamil	Yes, always	English, Tamil	No	Not shown
	Bedrulenka 2012	India	U/A	23	Start only	Telugu, English	Yes, always	Telugu	No	Not shown
	Satyaprem ki Katha	India	U/A	4	Start only	English, Hindi	Yes, always	Hindi	No	Not shown
	Her	India	U/A	6	Start only	Telugu, English	Yes, always	Telugu	No	Not shown

**Table 2:** Compliance with OTT (over-the-top) rules, 2023, in top web series in India U: Unrestricted public exhibition, suitable for all ages; UA: Unrestricted public exhibition but with caution to give parental guidance for those under 12; A: Only adults are allowed to view these programs.

OTT platform	Name of the web series	Country of production	Viewer category	Tobacco incidents	A-V disclaimer		Anti-tobacco static message		Anti-tobacco health spots	
					Shown	Language	Shown	Language	Shown	Language
MX Player	Familiar Wife	Korea	U/A	0	No	-	No	-	No	-
	Moonlight Romance	China	U/A	0	No	-	No	-	No	-
	Nishabd	India	U/A	5	Start only	Hindi	Yes, once	Hindi	No	Not shown
	Campus Diaries	India	U/A	0	Start only	English	No	-	No	-
	Flames of Fate	Turkey	U/A	0	No	-	No	-	No	-
Jio Cinema	Kaalkot	India	A	2	No	Not shown	No	Not shown	No	Not shown
	The Real Housewives of Beverly Hills	US	U/A	0	No	-	No	-	No	-
	Succession	US	A	2	No	Not shown	No	Not shown	No	Not shown
	Game of Thrones	US	A	0	No	-	No	-	No	-
	Bigg Boss OTT S2	India	U/A	0	No	-	No	-	No	-
Hotstar	Aakhri Sach	India	A	4	No	Not shown	No	Not shown	No	Not shown
	The Freelancer	India	U/A	1	No	Not shown	No	Not shown	No	Not shown
	Kaala	India	A	13	Start only	English	No	Not shown	No	Not shown
	Grey's Anatomy	US	U/A	0	No	-	No	-	No	-
	How I Met Your Mother	US	U/A	0	No	-	No	-	No	-
	Modern Family	US	U/A	0	No	-	No	-	No	-
Netflix	One Piece	UK, US, Japan	U/A	0	No	-	No	-	No	-
	Guns & Gulaabs	India	U/A	27	Start only	English	No	Not shown	No	Not shown
	Kengan Ashura	Japan	A	4	No	Not shown	No	Not shown	No	Not shown
	Jujutsu Kaisen	Japan	U/A	0	No	-	No	-	No	-
	Sex Education	UK	A	0	No	-	No	-	No	-
SonyLiv	Scam 1992: The Harshad Mehta Story	India	A	5	Start only	English	No	Not shown	No	Not shown
	Charlie Chopra & the Mystery of Solang Valley	India	U/A	5	Start only	English	Yes, always	English	No	Not shown
	Kavya	India	U/A	0	No	-	No	-	No	-
	Scam 2003: The Telgi Story	India	A	10	Start only	English	Yes, always	English	No	Not shown
	K.B.C. 2023	India	U/A	0	No	-	No	-	No	-
Zee5	Abar Prolay	India	A	5	Start only	English	No	Not shown	No	Not shown
	Kashmir Files: Unreported	India	A	0	Start only	English	No	-	No	-
	Code M	India	A	14	Start only	English	No	Not shown	No	Not shown
	Taj	India	U/A	3	Start only	English	No	Not shown	No	Not shown
	Duranga	India	U/A	0	Start only	English	No	-	No	-

**Table 3:** Summary of Tobacco Imagery Incidence and Compliance with OTT Rules, 2023, by popular movies and web series in select OTT (over-the-top) platforms as of September 26, 2023 U: Unrestricted public exhibition, suitable for all ages; UA: Unrestricted public exhibition but with caution to give parental guidance for those under 12; A: Only adults are allowed to view these programs.

OTT show characteristics		Tobacco incidence (number of times) (n=60)			A-V disclaimer shown (n=60)		Anti-tobacco health spots shown (n=60)		Anti-tobacco static message shown at the time of tobacco image incidence (n=28)		
		Nil	<9	> 9	No	Start only	No	Start only	No	Once	Always
All n (%)		32 (53.4)	19 (31.6)	9 (15)	36 (60)	24 (40)	59 (98.3)	1 (1.7)	17 (60.7)	1 (3.5)	10 (35.7)
Show type	Movies	15 (51.7)	9 (31)	5 (17.3)	17 (58.6)	12 (41.4)	28 (96.6)	1 (3.4)	6 (42.9)	0 (0)	8 (57.1)
	Web series	17 (54.8)	10 (32.3)	4 (12.9)	19 (61.3)	12 (38.7)	31 (100)	0 (0)	11 (78.6)	1 (7.1)	2 (14.3)
OTT platform	Amazon Prime	1 (20)	2 (40)	2 (40)	1 (20.0)	4 (80.0)	5 (100)	0 (0)	0 (0)	0 (0)	4 (100)
	Hotstar	6 (60)	3 (30)	1 (10)	8 (80.0)	2 (20.0)	9 (90)	1 (10)	3 (75)	0 (0)	1 (25)
	Jio Cinema	5 (50)	3 (30)	2 (20)	9 (0.0)	1 (10)	10 (100)	0 (0)	3 (60)	0 (0)	2 (40)
	MX Player	9 (90)	1 (10)	0	8 (80)	2 (20)	10 (100)	0 (0)	0 (0)	1 (100)	0 (0)
	Netflix	5 (50)	3 (30)	2 (20)	6 (60)	4 (40)	10 (100)	0 (0)	5 (100)	0 (0)	0 (0)
	SonyLiv	2 (40)	2 (40)	1 (20)	2 (40)	3 (60)	5 (100)	0 (0)	1 (33.3)	0 (0)	2 (66.7)
	Zee5	4 (40)	5 (50)	1 (10)	2 (20)	8 (80)	10 (100)	0 (0)	5 (83.5)	0 (0)	1 (16.7)
Country of production	Foreign	21 (87.5)	3 (12.5)	0	24 (100)	0 (0)	24 (100)	0 (0)	3 (100)	0 (0)	0 (0)
	India	11 (30.6)	16 (44.4)	9 (25)	12 (33.3)	24 (66.7)	35 (97.2)	1 (2.8)	14 (56)	1 (4)	10 (40)
Viewer category	A	7 (41.1)	7 (41.1)	3 (18)	10 (58.8)	7 (41.1)	17 (100)	0 (0)	16 (94.1)	0 (0)	1 (5.8)
	U/A	24 (57.1)	12 (28.5)	6 (14.2)	25 (59.5)	17 (40.4)	41 (97.6)	1 (2.3)	32 (76.1)	1 (2.3)	9 (21.4)
	U	1 (100)	0 (0)	0 (0)	1 (100)	0 (0)	1 (100)	0 (0)	1 (100)	0 (0)	0 (0)
Tobacco incidence	Nil	-	-	-	28 (87.5)	4 (12.5)	32 (100)	0 (0)	-	-	-
	1–9 times	-	-	-	7 (36.8)	12 (63.2)	18 (94.7)	1 (5.3)	13 (68.4)	1 (5.3)	5 (26.3)
	>9 times	-	-	-	1 (11.1)	8 (88.9)	9 (100)	0 (0)	4 (44.4)	0 (0)	5 (55.6)
Language	Hindi	0 (0)	10 (83.3)	2 (16.7)	0 (0)	4 (100)	0 (0)	1 (100)	0 (0)	1 (25)	3 (75)
	Others	3 (8.3)	17 (47.2)	16 (44.4)	0 (0)	22 (100)	0 (0)	0 (0)	0 (0)	0 (0)	8 (100)

In the included shows, 32 had no incidences of tobacco imagery, and 28 had ten or more tobacco imagery incidences. Audio-visual disclaimers and anti-tobacco health spots were not shown in 60% and 98.3% of cases, respectively. Only 40% of shows had an AV disclaimer at the start of the show, and no show had an AV disclaimer in the middle of the show. Only one show had anti-tobacco health spots at the beginning of the show, and no show had health spots at the middle of the show. In 28 shows where tobacco imagery was shown, 35.7% of shows always showed static warning messages, and others did not comply with the legal provision on static messages.

The incidence of tobacco imagery in movies and web series was 48.3% and 45.2%, respectively. Tobacco incidences among popular shows streamed on Amazon Prime (80%), SonyLiv (60%), and Zee5 (60%) were higher than on JioCinema (50%), Netflix (50%), Hotstar (40%), and MX Player (10%) platforms. Indian-produced shows had a higher incidence of tobacco imagery than those of non-Indian production. Among the viewers' category, i.e., A, U/A, and U had tobacco incidences of 59%, 42.7%, and 0%, respectively. None of the movies and web series, irrespective of age rating, country of production, or OTT platform, fully complied with the AV disclaimer and health spot provisions of the OTT Rules, 2023. Only ten out of 28 had fully complied with the Rule's static health warning messages provision, and it was higher among movies (57.1%) than web series (14.3%). All the shows from Amazon Prime only fully complied with static warning message provisions. None of the foreign production shows complied with the static warning message provision, while 2-in-5 shows of Indian production fully complied with the provision. In the U/A viewer category show, the static warning message was fully complied with 21.4%. Shows with higher tobacco incidences also had higher compliance with static warning message provisions. The disclaimer was shown in either Hindi (100%) or a combination of a regional language and English (100%). The health spots, when presented, were always in Hindi (100%). The static message during tobacco imagery was always shown in one of India's regional languages, Hindi (75%), English, or both (100%).

## Discussion

This study observed a total of 237 tobacco imagery incidents in 60 movies and web series, averaging about 3.95 incidences per popular show on OTT platforms in India. Nearly half of the movies and web series have at least one instance of tobacco imagery. About 3.4% of movies and none of the web series complied with the anti-tobacco health spot provisions. Approximately two in five movies and web series comply with the audio-visual disclaimer provisions of the OTT Rule 2023. Among 28 movies and web series that depicted tobacco images, 57% and 14%, respectively, had fully complied with static warning message provisions. Inconsistency in executing anti-tobacco measures persists among diverse platforms and content regarding language and production origin. However, the overall adherence to regulations is lower and more unbalanced than desired. While age ratings indicate suitability for audiences above 13 or 16, they do not necessarily ensure compliance with specific regulations related to the display of tobacco imagery.

A study by Kulkarni et al. [26] in 2020 suggested that 34 out of 47 (72%) movies had tobacco imagery incidents. This study found 137 instances of tobacco imagery in 14 out of 29 (48%) movies, indicating a decrease in the incidence of tobacco images in movies. The study also recorded that 29% of movies with tobacco imagery had anti-tobacco health spots in the beginning, and 15% of movies had health spots midway. In this study, there were no health spots in the middle, and only 7% of the movies with tobacco imagery had a health spot in the beginning, indicating much lower compliance by OTT platforms compared to film theatres. Movies on OTT platforms lack a defined middle interval, which could account for the absence of health spots in the middle.

Compared to Kulkarni et al.'s findings [26], this study has fewer instances of health spots despite the OTT Rule 2023 being in force, which is concerning given its popularity among the youth. Kulkarni et al. also found that 24% of movies with tobacco imagery had an audio-visual disclaimer at the beginning and none in the middle. In this study, 86% of movies with tobacco imagery had an audio-visual disclaimer at the beginning and none in the middle, indicating an improvement in showing an audio-visual disclaimer at the beginning of movies by OTT platforms. Kulkarni et al. also noted that 87% of the movies with tobacco imagery displayed static warning messages, but most (92%) were non-compliant with the COTPA provisions. In this study, eight (57.1%) movies with tobacco imagery displayed static anti-tobacco warning messages at the time of the display of tobacco imagery on the screen. Despite the observed lesser adherence to static warnings, these OTT platforms were found to comply with the broader regulations that had been notified.

A study by Arora et al. (2021) suggested that 70% (from 10 web series) of the popular shows on these platforms among young adults featured tobacco usage [11]. In this study, 14 out of 31 web series had tobacco imagery (45%), indicating fewer incidences of tobacco imagery. Arora et al. (2021) also found that not a single episode in any of the seven seasons of these shows, not even the ones with historical characters, had an audio-visual disclaimer regarding the harmful effects of tobacco use or anti-tobacco static warning messages or health ads. In this study, 38% of web series had an audio-visual disclaimer, none had anti-tobacco health spots, and 21% of web series had an anti-tobacco static message, suggesting limited adherence to the OTT Rules of 2023 by the OTT platforms. Non-compliance of these popular web series with the anti-tobacco health spots requirements is again concerning.

Alfayad et al. [27], in 2021, revealed that out of 479 intervals, 129 intervals (26.9%) of Amazon and Netflix OTT contents in the United Kingdom had tobacco incidents. In this study, five-minute interval-wise data were not taken, but the 137 incidences of tobacco imagery in 12 movies represent a relatively high incidence. According to a 2018 Truth Initiative survey [28], smoking is depicted in 79% of the on-demand streaming shows that young Americans between the ages of 15 and 24 find appealing. In this study, 52% of the movies and web series had tobacco imagery, indicating a slight improvement in restricting tobacco imagery by OTT platforms in their movies and web series.

Nazar et al. [29] investigated the impact of regulations on tobacco depictions in top-grossing Bollywood films from 2006 to 2017. Their analysis revealed that out of 240 movies, 19% had a U rating, 68% had a U/A rating, and 14% had an A rating. The compliance rates were 22% for audio-visual disclaimers, 33% for anti-tobacco health spots, and 31% for on-screen static warnings during the on-screen display of tobacco imagery. In contrast, the current study focused on 60 movies and web series, with 28.3% having an A rating, 70% with a U/A rating, and 1.67% with a U rating. The current study concentrated on top movies and web series available on OTT platforms as of September 26, 2023. Unlike Nazar et al., who assessed tobacco depictions annually, this study provides a snapshot of compliance in a specific timeframe. The compliance rates in our study differed, with 40% for audio-visual disclaimers, 1.7% for anti-tobacco health spots, and 39.2% for on-screen static warnings during tobacco use. This indicates the OTT platforms are more likely to display the audio-visual disclaimer at the beginning of the movie and web series but remain non-compliant with the prescribed anti-tobacco health spots.

In the current OTT content, as far as movies and web series are concerned, full compliance with OTT Rules 2023 is limited. Since the revised Rule came into effect on September 1, 2023, there is a need to sensitize OTT platforms to the new rules and the importance of the same for public health. Earlier studies have also suggested minimal compliance with COTPA provisions in Indian movies and television shows [11, 26]. Since OTT platform content on mobile is privately viewed by individuals, it is important to protect youths from tobacco promotion through OTT content. Among the provisions, there is a complete violation of health spot and audio-visual warning at the middle of the show, which needs policymakers' attention and a definite guideline to identify the middle of the show by considering the indicated duration of the show in minutes on the respective OTT platforms.

Another important point is that OTT content is available from countries of origin outside India, and such producers or creators of online curated content may not be aware of the Indian OTT Rules. Therefore, OTT platforms must take utmost care to ensure compliance with Indian Laws when such producers or creators choose to publish their content on their platforms. Another issue is the language of health spots, audio-visual warnings, and static messages, which have been highlighted in previous studies as well [26, 29]. In current times, OTT platforms are airing content in multiple languages with or without subtitles. This makes the situation complex to identify the most suitable language for the disclaimers, health spots, or warning messages. Here, OTT platforms or publishers of online content must ensure that a viewer’s discretion in selecting the language must by default result in the same language for the disclaimers, health spots, audio-visual warnings, or static messages to achieve the objectives of the OTT Rules.

The study has several strengths. First, the top 10 movies and web series from select popular OTT platforms were chosen based on viewership and feasibility. The top 10 list was chosen to capture popular content and audience preferences. This strategy enabled a methodical and controllable study of a wide variety of content from popular OTT platforms consumed by most audiences. Second, the trained coder's data was verified by senior authors. Thus, the data presented here has fewer errors. Third, this study was conducted after the new rule came into force; thus, it captured compliance with the OTT Rules, 2023, and has the potential to inform law enforcement agencies regarding levels of compliance and necessary remedial actions from them. The public health implication of this study lies in the level of compliance of OTT shows, which is vital for comprehensive tobacco control measures in India.

The study has several limitations. The selection of OTT platforms was based on subscriber numbers released by non-government agencies [21-24], and authenticity couldn’t be verified. Nevertheless, the selected OTT platforms have regularly featured in top OTT platform lists in the country. The OTT platforms do not maintain a top 10 list in a uniform manner, which had a chance to affect the selection of the number of movies and web series. After selection, the movie "Olympus Has Fallen" in MX Player was removed, and data couldn’t be collected though the top 6th was replaced for the movie. Similarly, two movies (The Expendables and Operation Red Sea) had been removed from the platform on the date of completion of manuscript writing, making it difficult to verify it in the latter stage of the study. The selected popular OTT platforms and their top 10 shows are very dynamic and keep on changing, which means the definition of "popular shows" and "popular OTTs" will vary with time and is applicable as of September 26, 2023.

## Conclusions

The study has three major conclusions. First, despite the challenges, several OTT platforms and online content providers were able to implement the new OTT Rules since September 1, 2023, although in a limited way and in a different form. Second, the incidence of tobacco imagery in movies, including those rated for minors, is still common on OTT platforms, requiring active and regular monitoring and enforcement. Third, despite the Government of India's announcement of strict action in cases of violation of the OTT Rules, 2023, most of the OTT platforms have violated the norms and failed to comply fully with the regulations. In view of the above, the inter-ministerial committee designated for enforcing the provisions of the Rules should take note of these violations and take appropriate action. The Ministry of Health and Family Welfare should consider providing necessary directions, including guidelines and orientation, for all stakeholders to better comply with the rules to protect the unsuspecting and vulnerable youth from unwarranted exposure to tobacco imagery on OTT platforms. Routine follow-up studies should be conducted at regular intervals to measure and monitor compliance with and mitigate challenges in the implementation of the important OTT Rule of 2023. The AI technology, when available, may also be used for real-time monitoring of tobacco imagery in OTT shows.

## Appendices

**G.S.R. 400(E)**. In exercise of the power conferred by section 31 of the Cigarettes and other Tobacco Products (Prohibition of Advertisement and Regulation of Trade and Commerce, Production, Supply and Distribution) Act, 2003 (34 of 2003), the Central Government hereby makes the following rules, further to amend the Cigarettes and Other Tobacco Products (Prohibition of Advertisement and Regulation of Trade and Commerce, Production, Supply and Distribution) Rules, 2004, namely:- 1. (1) These rules may be called the Cigarettes and other Tobacco Products (Prohibition of Advertisement and Regulation of Trade and Commerce, Production, Supply and Distribution) Amendment Rules, 2023. (2) They shall come into force on and after the expiration of three months from the date of their publication in the Official Gazette. 2. In the Cigarettes and other Tobacco Products (Prohibition of Advertisement and Regulation of Trade and Commerce, Production, Supply and Distribution) Rules, 2004, after rule 10, the following rule shall be inserted, namely. - “11. Health spots, message and disclaimer in online curated contents of tobacco products by the publisher. (1) Every publisher of online curated contents displaying tobacco products or their use shall.- (a) display anti-tobacco health spots, of minimum thirty seconds duration each at the beginning and middle of the programme; (b) display anti-tobacco health warning as a prominent static message at the bottom of the screen during the period of display of the tobacco products or their use in the programme; (c) display an audio-visual disclaimer on the ill-effects of tobacco use, of minimum twenty seconds duration each, in the beginning and middle of the programme; (2) The health spots, message and disclaimer shall be made available to the publisher of the online curated content on the website “mohfw.gov.in” or “ntcp.mohfw.gov.in”

(3) The anti-tobacco health warning message as specified in clause(b) of sub-rule (1) shall be legible and readable, with font in black colour on white background and with the warnings "Tobacco causes cancer" or "Tobacco kills".

(4) The anti-tobacco health warning message, health spot and audio-visual disclaimer, shall be in the same language as used in the online curated content.

(5) The display of tobacco products or their use in online curated content shall not extend to -

(a) display of the brands of cigarettes or other tobacco products or any form of tobacco product placement;

(b) display of tobacco products or their use in promotional materials.

**Explanation**. For the purposes of this rule “programme” means online curated audio-visual content.

(6) If the publisher of online curated content fails to comply with the provisions of sub-rules (1) to (5), an interministerial committee consisting of representatives from the Ministry of Health and Family Welfare, Ministry of Information and Broadcasting and Ministry of Electronics and Information Technology, shall take action suo motu or on a complaint, and after identifying the publisher of online curated content, issue notice giving reasonable opportunity to explain such failure and make appropriate modification in the content.

**Explanation**. For the purpose of this rule,

a. the expression “online curated content” means, any curated catalogue of audio-visual content, other than news and current affairs content, which is owned by, licensed to, or contracted to be transmitted by a publisher of online curated content, and made available on demand, including but not limited through subscription, over the internet or computer networks, and includes films, audio visual programmes, documentaries, television programmes, serials, series, podcasts and other such content;

b. the expression “publisher of online curated content” means, a publisher who, performing a significant role in determining the online curated content being made available, makes available to users a computer resource that enables such users to access online curated content over the internet or computer networks, and such other entity called by whatever name, which is functionally similar to publishers of online curated content but does not include any individual or user who is not transmitting online curated content in the course of systematic business, professional or commercial activity.
